# Involuntary Monitoring of Sound Signals in Noise Is Reflected in the Human Auditory Evoked N1m Response

**DOI:** 10.1371/journal.pone.0031634

**Published:** 2012-02-28

**Authors:** Lothar Lagemann, Hidehiko Okamoto, Henning Teismann, Christo Pantev

**Affiliations:** 1 Institut für Biomagnetismus und Biosignalanalyse, Westfälische Wilhelms-Universität Münster, Münster, Germany; 2 Department of Integrative Physiology, National Institute for Physiological Sciences, Okazaki, Japan; Baycrest Hospital, Canada

## Abstract

Constant sound sequencing as operationalized by repeated stimulation with tones of the same frequency has multiple effects. On the one hand, it activates mechanisms of habituation and refractoriness, which are reflected in the decrease of response amplitude of evoked responses. On the other hand, the constant sequencing acts as spectral cueing, resulting in tones being detected faster and more accurately. With the present study, by means of magnetoencephalography, we investigated the impact of repeated tone stimulation on the N1m auditory evoked fields, while listeners were distracted from the test sounds. We stimulated subjects with trains of either four tones of the same frequency, or with trains of randomly assigned frequencies. The trains were presented either in a silent or in a noisy background. In silence, the patterns of source strength decline originating from repeated stimulation suggested both, refractoriness as well as habituation as underlying mechanisms. In noise, in contrast, there was no indication of source strength decline. Furthermore, we found facilitating effects of constant sequencing regarding the detection of the single tones as indexed by a shortening of N1m latency. We interpret our findings as a correlate of a bottom-up mechanism that is constantly monitoring the incoming auditory information, even when voluntary attention is directed to a different modality.

## Introduction

It is a vital capability of the human auditory system to detect and track sounds emitted by potentially important sources. In most cases, an acoustic signal reaching the ear is a mixture of sounds emitted by several different objects or events. One of the most crucial tasks for the auditory system is thus to separate sounds coming from distinct sources and to keep track of them. Sound features that distinguish sources from each other are e.g. spectrum, intensity, or phase [1]. In previous behavioral studies it was shown that it is easier for subjects to detect tones if these are cued by the context presented beforehand [2]–[5]. Cues can be e.g. auditory stimuli that have the same pitch as the target stimulus, stimuli that have a fixed pitch relation to the target [2], or patterns that allow conclusions about the pitch of the target [4]. Besides the fact that a tone can cue a subsequent tone of the same frequency, it is well established that the repetitive presentation of the same tone can lead to a decrease of the neural response to that tone [6]–[10].

In a previous study [11], we presented subjects either constantly the same frequency, or randomly changing frequencies while the subjects' attention was directed away from the auditory input towards the visual modality. The tones were either presented in silence or embedded in noise. While in the “constant sequencing condition” stimuli were cued by the preceding tone of the same frequency, this was not the case in the “random sequencing condition”, in which the stimuli alternated randomly. In the silent condition, we found differences in N1m source strength depending on the sequencing mode, with less activation in the constant sequencing condition than in the random sequencing condition. We attributed this finding to habituation and/or refractory mechanisms acting differently depending on the type of sequencing. Moreover, we found that when the tones were presented in noise, the average latency in the constant sequencing condition was significantly shorter compared to random sequencing, which we attributed to cueing mechanisms. The results indicated that background noise has a strong impact on cueing and mechanisms of habituation and/or refractoriness and that this impact is displayed in the N1m auditory evoked field originating in the human auditory cortex.

The goal of the present study was to address two general issues: First, we investigated the time course of N1m source strength in silence and noise. Second, we attempted to determine the number of repetitions of the same stimulus that are needed to establish and stabilize a sequencing effect with regard to N1m latency. To study these matters, subjects were presented with sound trains composed of four tones of either the same frequency or of randomly changing frequencies. The trains were presented either in silence or embedded in noise. We hypothesized that in silence we would find differences regarding source strength decline between sequencing conditions. Concerning the latencies, we expected differences between sequencing conditions to occur in the noisy condition.

## Methods

### Stimuli and experimental design

**Figure 1:**
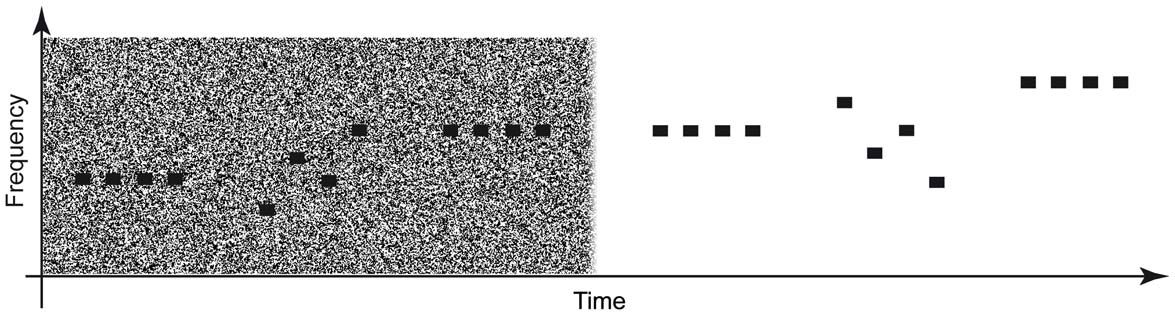
Schematic depiction of the stimulation. On the x- and y-axis, time and frequency are indicated. Black bars represent individual tones (duration = 500 ms). Blocks of noisy and silent backgrounds were presented alternately. Each block consisted of ten trains of four tones in random sequencing and ten trains of four tones of constant sequencing that were randomly distributed across the whole block.

40 Hz amplitude-modulated tones (modulation depth 100%) of eight different carrier frequencies (250, 450, 700, 1000, 1370, 1850, 2500, 3400 Hz) with a duration of 500 ms were used as test stimuli (TS). The stimuli were concatenated to stimulus trains of 4 items. The trains consisted either of tones of identical frequency (constant sequencing condition) or of tones randomly chosen from the eight frequencies (random sequencing condition). The randomization was controlled to ensure that the same tone could appear at maximum two times in a row within a random train. In each sequencing (constant vs. random) and noise (noise vs. silence) condition 96 trains were presented, amounting to 382 trains in total. The stimuli were prepared using MATLAB (The MathWorks Inc.) and CoolEdit (Syntrillium). The inter-stimulus-interval (ISI) was fixed to 500 ms, and the inter-train-interval (ITI) was randomized between 2.5 and 3.5 s, resulting in an average ITI of 3 s. In the noise condition, 8000 Hz low-pass filtered white noise was added to the stimuli. The total root mean square (RMS) intensity of the noise was 10 dB above stimulus intensity. The noise blocks were linearly faded in and out for 50 ms. Each run consisted of alternating blocks of different signal-to-noise ratios (i.e. noise vs. silence), containing trains of constant sequencing and trains of random sequencing that were succeeding randomly. An idealized depiction of the stimulation is shown in Figure 1.

We used Presentation (Neurobehavioral Systems, Albany, CA, United States) to control the timing of sound presentation, and SRM-212 electrostatic earphones (Stax, Saitama, Japan) to transduce sound stimuli. All sounds were presented diotically through silicon tubes (length: 60 cm; inner diameter: 5 mm) and silicon earpieces adjusted to individually fit into each subject's ears. Before starting the magnetoencephalography (MEG) acquisition, each subject's hearing threshold for the 1000 Hz carrier frequency TS was measured for each ear. During the MEG session, the tonal stimuli were presented at an intensity of 40 dB above this individual threshold. During stimulus presentation, subjects were watching a silent movie, and after each of the six runs questions regarding the content of the movie were asked, thus ensuring that attention had been directed to the visual domain and was therefore distracted from the auditory modality.

We tested 16 healthy subjects (age 22–30 years), 9 of which were females. All subjects had normal hearing and were right handed as assessed with the Edinburgh Handedness Inventory [12]. All subjects were fully informed about the study and gave written consent for their participation. The study was approved by the Ethics Commission of the Medical Faculty of the University of Münster and conformed to The Code of Ethics of the World Medical Association (Declaration of Helsinki).

### Data acquisition and analysis

The auditory evoked fields were recorded with a whole-head 275 channels MEG system (Omega; CTF Systems, Coquitlam, British Columbia, Canada) in a magnetically shielded and acoustically silent room. Subjects were instructed not to move their head and were monitored by means of video camera by the experimenter. The magnetic fields were digitized with a sampling rate of 600 Hz. The magnetic fields evoked by each tone were averaged for each signal-to-noise condition (silence and noise), sequencing condition(random and constant), and tone position (1^st^, 2^nd^, 3^rd^, and 4^th^), starting 200 ms prior to TS-onset, and ending 800 ms after TS-onset, applying a 1–20 Hz band-pass filter and baseline correction relative to the 100 ms pre-stimulus interval. Epochs containing field changes larger than 2.5 pT were rejected as artifact epochs.

We evaluated the auditory evoked fields with regard to the N1m response. Since MEG sensors have very little sensitivity to purely radially oriented sources, the N1m basically has its origin in neuronal currents that have a component oriented tangentially to the skull [13], [14] and is thought to reflect mainly temporal lobe activity [15]. For the source localization of the N1m response, the auditory evoked fields across all conditions of the first run were averaged. Then, the N1m response was identified as the time point of maximal RMS value of the global field amplitude around 150 ms after TS-onset. A 10 ms interval around this N1m peak latency was selected, and the source locations and orientations were estimated by single equivalent current dipole modeling (one dipole for each hemisphere) for each subject individually [16]. As source space we used a spherical head model derived from anatomical magnetic resonance images (MRIs) of each subject. Dipole estimations with an error rate exceeding 10% (i.e. a goodness of fit lower than 90%) were excluded from further analysis, reducing the number of subjects to n = 12. The mean goodness of fit for the dipoles of the subjects who were not rejected was 96.7%. The estimated sources were fixed in location and orientation for each hemisphere of each subject as a spatial filter [17]. Using this spatial filter, source waveforms of the averaged auditory evoked fields from each run were calculated for each respective condition. The obtained source waveforms were then averaged across all six runs for each condition and hemisphere. The average of the peaks with the highest source strength for each hemisphere in each condition in the time range between 90 and 220 ms was used for further statistical analysis. N1m source strength and latency elicited by the TS were analyzed separately for each noise condition via repeated-measures analyses of variance (ANOVA) using two factors: SEQUENCING (constant vs. random), and TONE-POSITION (position 1–4). Before calculating the ANOVAs, Mauchly's test was conducted. In cases where sphericity of the data was not given, we report Greenhouse-Geisser corrected values. Predicted differences between tone pairs were tested via planned comparisons. Differences between condition pairs for which we did not have a priori hypotheses were investigated using Bonferroni-Holm corrected post-hoc tests [18].

## Results

**Figure 2:**
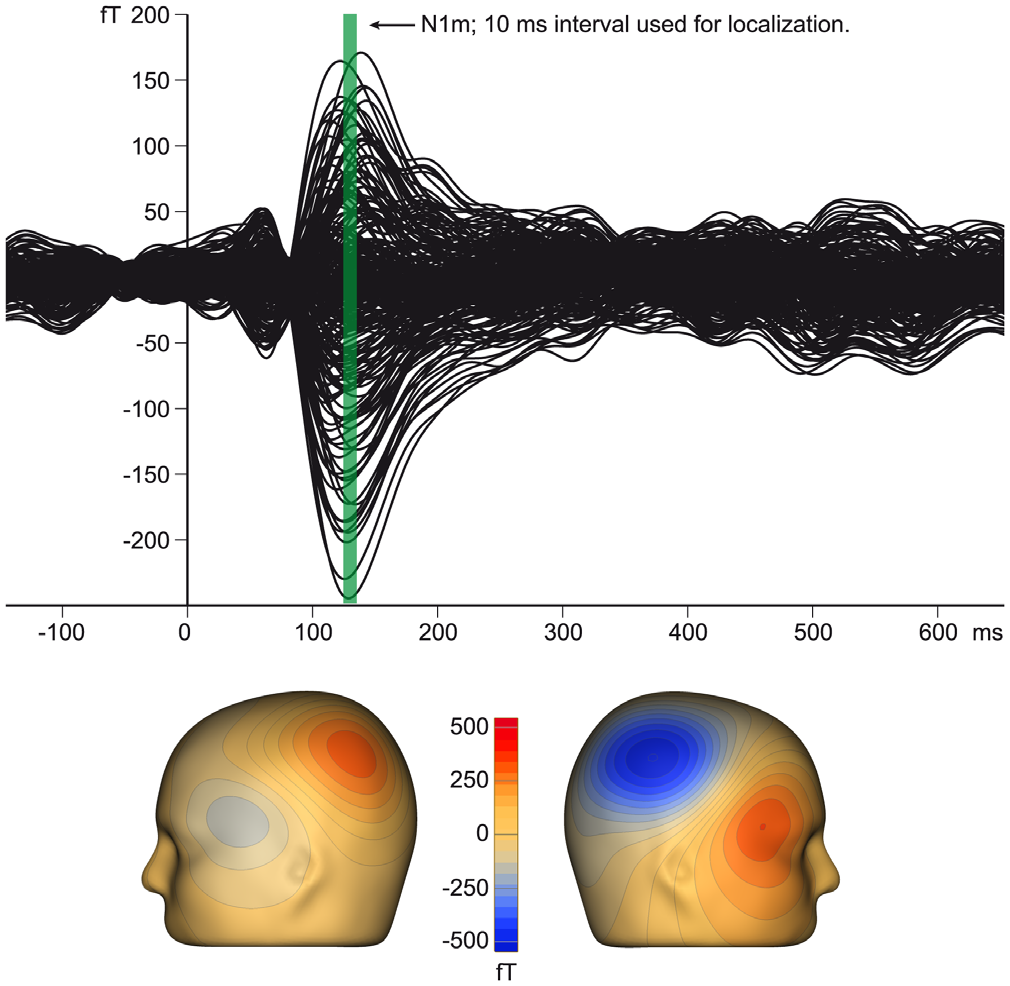
Individual auditory evoked field data. Top: response averaged across all stimuli presented in the first run; an articulate N1m peak is discernible. The green area indicates the 10 ms time range around the latency of highest RMS field amplitude of all sensors. This time range was taken for source reconstruction. Bottom: the magnetic flux at 130 ms, the point of highest RMS field amplitude demonstrates clear dipolar field distribution.

**Figure 3:**
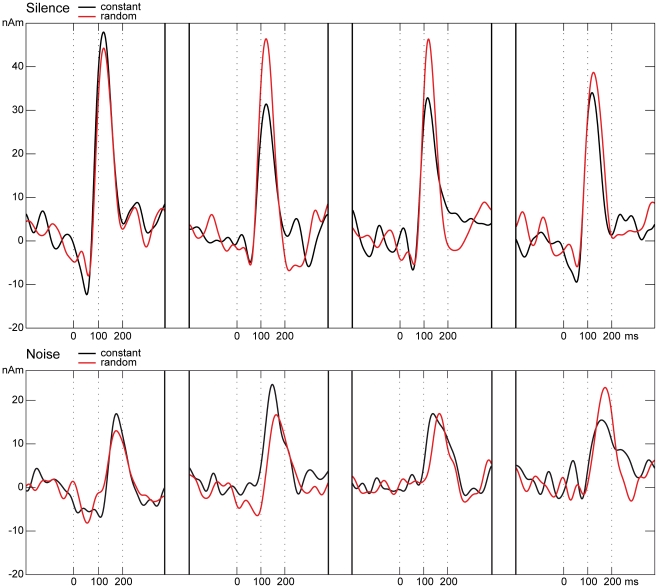
Individual source wave forms of the N1m responses. The x-axis shows the time in ms related to stimulus onset. On the y-axis source strength in nAm is denoted. Upper panel: source wave forms to tone trains presented in silence in the constant condition (black line) and in the random condition (red line). Bottom: source wave forms to tone trains presented in noise (color coding same as in the upper panel).

Sensor space data of a representative subject is depicted in Figure 2. Figure 3 shows the individual source wave forms of the N1m responses of the same subject to stimulus trains within the different conditions.

### Source strength

**Figure 4:**
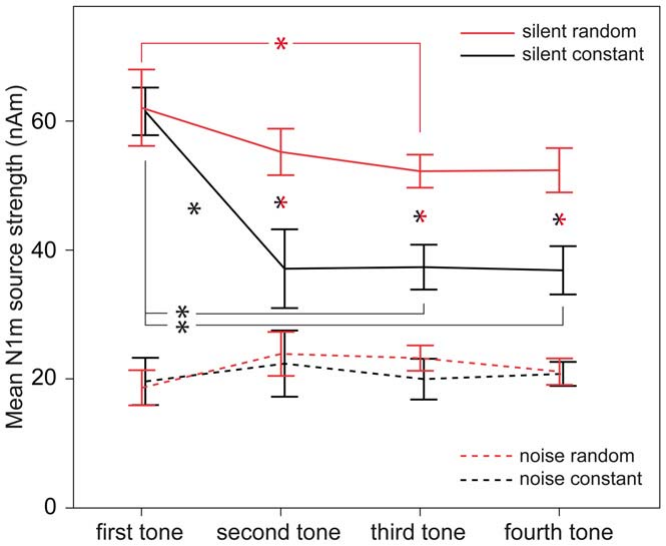
N1m source strength in each condition. The x-axis depicts the respective tone positions. The y-axis denotes the mean N1m source strength. Red color symbolizes the random condition, and black the constant sequencing condition. Values in the silent condition are drawn with solid lines, and values in the noise condition are symbolized by dashed lines. Single colored asterisks mark significant differences between tones of the same sequencing condition. Double colored asterisks mark differences between tones of different sequencing conditions in the same respective position. Error-bars denote the 95% confidence interval limits of the arithmetic mean across subjects.

The interaction plots for N1m source strength are shown in Figure 4. In silence a clear drop in source strength can be seen between first and second tone in the constant sequencing condition, but not in the random sequencing condition. To check for possible main effects and interactions, we conducted an ANOVA with the factors SEQUENCING (constant vs. random) and TONEPOSITION (position 1–4) for silent and noisy backgrounds separately. For the noisy background, the ANOVA yielded neither significant main effects nor interactions (SEQUENCING (F(1,11) = 1.102, n.s.), TONE-POSITION(F(1.69,18.59) = 2.52, n.s.), SEQUENCING×TONEPOSITION (F(3,33) = 0.598, n.s.)). Since the ANOVA did not yield any significant results, and since we did not predict any differences in this condition, we did not further analyze these data. The ANOVA for the silent surrounding showed the following: SEQUENCING (F(1,11) = 54.991, p<0.001), TONE-POSITION (F(1.425,15.683) = 23.456, p<0.001), SEQUENCING×TONE-POSITION (F(1.699,18.690) = 14.654, p<0.001).

Due to the repeated presentation of the same stimulus in the constant sequencing condition, we expected significant differences between the first tone and each of the following three tones. Planned comparisons showed this difference: the source strength drop between first and second tone reached significance (t(11) = 5.532, p<0.001), and also first and third (t(11) = 10.061, p<0.001) and first and fourth tone (t(11) = 8. 079, p<0.001) differed significantly. We further expected larger source strength values for the tones at positions two to four in the random sequencing condition compared to the tones at the same respective positions in the constant sequencing condition. Again, planned comparisons revealed that the values differed significantly (tone pair position 2: t(11) = −5.612, p<0.001; tone pair position 3: t(11) = −7.894, p<0.001; tone pair position 4: t(11) = −6.455, p<0.001).). For the remaining contrasts we did not have hypotheses, thus further source strength differences were evaluated using Bonferroni-Holmes corrected post-hoc tests. The decrease of source strength from first to second tone in the random sequencing condition was not significant (t(11) = 1.817, n.s.). The source strength decline from the first tone was significant at the third tone (t(11) = 4.669, p<0.01). The difference between the first and the fourth tone in the random sequencing condition was not significant (t(11) = 2.752, n.s.). Source strength values between the second and the third as well as between the third and the fourth tone did not differ significantly. This held true for the constant sequencing condition (second to third: t(11) = −0.81, n.s.; third to fourth: t(11) = 0.293, n.s.) as well as for the random sequencing condition (second to third: t(11) = 1.309, n.s.; third to fourth: t(11) = −0.63, n.s.).

### Latency

**Figure 5:**
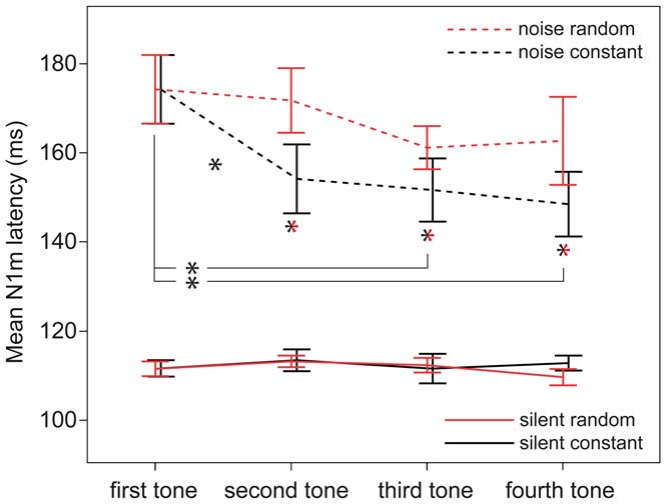
N1m latencies in each condition. N1m latency values in milliseconds are displayed on the y-axis. The x-axis shows the respective tone positions. Figure legend according to Figure 4.

N1m latency is visualized in Figure 5. Obvious differences show between the first and the second, the first and the third and the first and the fourth tone in the constant sequencing condition in noise. The ANOVA for the silent condition did not show any significant main effects for SEQUENCING (F(1,11) = 5.22, n.s.) or for TONEPOSITION (F(3,33) = 1.286, n.s.). Also, there was no significant interaction between SEQUENCING and TONEPOSITION (F(3,33) = 2.465, n.s.). Due to the lack of significant results in the ANOVA, and since we did not expect latency differences between sequencing conditions in silence, we did not further examine latency values elicited by tones presented in silence. The ANOVA for the N1m peak latency values in noise showed significant effects for all factors and interactions: SEQUENCING: (F(1,11) = 29.144, p<0.001), TONEPOSITION: (F(3,33) = 7.913, p<0.001), and SEQUENCING×TONEPOSITION: (F(3,33) = 2.897, p = 0.05).

We predicted latency differences between sequencing conditions in two tone pairs: We expected the cueing effect to set in latest at the fourth tone in the constant sequencing condition, but not in the random sequencing condition. Thus, we expected the N1m latency to the fourth tone in the constant sequencing condition to be shorter than the N1m to the respective tone in the random sequencing condition. Accordingly, we also expected tone four in the constant sequencing condition to differ significantly from tone one. The conducted planned contrasts showed the expected differences between those tones: tone four constant vs. tone four random t(11) = −2.603, p<0.05; tone one constant vs. tone four constant t(11) = 4.182, p<0.01. To further investigate latency differences in noise, we performed post-hoc tests for remaining 11 pairs of interest. The reported p-values are Bonferroni-Holms corrected. N1m latency dropped significantly from the first to the second tone (t(11) = 3.480, p<0.05) in the constant sequencing condition. In the constant sequencing condition also the latency difference between the first and the third tone was significant (t(11) = 5.079, p<0.01). In the random sequencing condition, the difference between the first and the second tone was not significant (t(11) = 0.788, n.s.). Also, the differences between the first and the third tone (t(11) = 2.706, n.s.) and between the first and the fourth tone did not reach significance (t(11) = 1.577, n.s.). There were no significant differences between the second and the third and the third and the fourth tone in any of the two sequencing conditions (constant: second vs. third t(11) = 0.427, n.s.; third vs. fourth t(11) = 0.575, n.s.; random: second vs. third t(11) = 2.267, n.s.; third vs. fourth t(11) = −0.355, n.s.). Comparing the respective tone positions two and three between sequencing conditions, we found significantly shorter latencies for the second (t(11) = −4.214, p<0.05)as well as for the third position(t(11) = −3.056, p<0.05) for tones presented in constant order.

## Discussion

We presented trains of four tones embedded either in noise or in a silent surrounding. The tones of a train were either of the same frequency or of randomly changing frequencies. Across the whole experiment, the tones presented to the subjects in each condition were identical. The tones only differed in the way they were sequenced within the trains. Several previous studies investigated the influence of noise on the detection of test stimuli and the influences of noise on N1m amplitude and latency. For this, mostly conditions with noisy background were compared to conditions without noise. While low levels of noise seem to enhance the N1m amplitude [19], high levels of noise consistently yield lower N1m amplitudes and longer peak latencies compared to the N1m elicited in a silent background [11], [20], [21]. Here, we did not investigate the influence of noise per se, but how stimulus sequencing and noisy background interact.

In the silent background, we did not find any differences regarding the latencies of the N1m to the tones with respect to sequencing condition or tone position. The N1m source strength on the other hand did differ depending on both, sequencing condition as well as tone position: In the constant sequencing condition, we found a sharp drop between first and second tone, with no further reduction thereafter. In the random sequencing condition, we found a more gradual decrease of source strength. While the difference between first and second tone was not significant in random sequencing, the difference between first and third tone was.

In the noisy surrounding, we did not find any differences in N1m source strength between the different sequencing conditions. The latencies of the N1m responses however did differ between sequencing conditions. In the constant sequencing condition, the latency dropped from first to second tone, but not any further for tones three and four. In the randomly sequenced stimulus trains, we could not find any significant differences between the tones.

Several previous studies have investigated the mechanisms driving response decline with repeated stimulation. The two explanations that are considered most are habituation and refractoriness. While habituation is thought to be a learning mechanism [9], refractoriness depends on the recovery cycles of the stimulated sensory cells [7], [8], [10]. The most obvious difference between refractoriness and habituation is the time course of response decline: habituation is characterized by an ongoing slow decrease of the response with repeated stimulation, while refractory mechanisms would elicit a fast drop of the response strength from the first to the second stimulation, but no further decline from the third stimulation on [8], [10]. While some studies reported evidence for habituation [9], [22], others found characteristics of refractoriness in the patterns of source strength decline with repeated stimulation [6], [8]. It has to be mentioned though, that the two mechanisms are not per se exclusive, and they may arise from different stages in the auditory processing pathway [10], [23].

Here, regarding the pattern of N1m source strength decline in the silent condition, we found a steep drop of source strength in the constant sequencing condition. After this drop, no further decline in source strength took place. This pattern thus seems to favor refractory mechanisms as suggested in previous studies [8], [10]. However, in the random sequencing condition we also found a drop in source strength. This drop was not significant from first to the second tone, but it was from the first to the third. Hence, the pattern reflects the gradual decrease usually associated with habituation. The reason that we did not see any indication of habituation in the constant condition might be due to complete “coverage” by the much stronger source strength decline resulting from refractory mechanisms.

An alternative approach might be to explain the decrease in source strength of the N1m by the mechanics of lateral/surround inhibition [24], [25]. This approach suggests that the stimulation by the first test tone excites a rather broad patch of neurons at and around the neurons with the best frequency. On the second presentation of the same tone solely the neural population tuned to the test tone frequency responds while the activity in the patch of adjacent neurons is suppressed. This narrowing down becomes apparent in the decrease of source strength since less neurons are activated than in the first instance of stimulation. Jääskeläinen and colleagues [24] suggest this mechanism as the basis for cueing. From our results it is not possible to tell apart surround inhibition from refractory mechanisms, since the source strength decline would look the same. Another finding though makes lateral/surround inhibition seem improbable in our case: May and colleagues [25] state after simulating and empirically validating a model on adaptation and lateral inhibition that their results “suggest that lateral inhibition on the cortical level is either strong but decays with a fast time constant (of the order of 100 ms) or that it is weak but decays slowly” (p.116). With an ISI of 500 ms our presentation rate was thus out of the time range in which lateral/surround inhibition is postulated to show its effects.

Our present results thus confirm the view that in the temporal range of our stimulation, habituation as well as refractory mechanisms are responsible for the source strength decline after repeated stimulation in silence. The findings support the hypothesis that in the short time range investigated, refractoriness has the larger impact compared to habituation [10]. When tones were presented against a noisy background, there were no significant source strength differences between different tone positions. It thus seems that the refractoriness of the sound-processing neurons caused by noise evened out any potential differences arising from repeated tonal stimulation or differential sequencing.

Several authors have found indications that the auditory N1m might be driven by at least two sources: a posterior source that peaks about 20–30 ms earlier than a more anterior source peaking accordingly later. It is also reported that the N1m to a rare stimulus gets more contribution from the anterior source than if it is played frequently or as a second tone after a first stimulus [26]–[28]. This might have played a role in our results but from our data we would not be able to tell a systematic source location difference. To do this we would have to perform a source localization for the N1m of every single frequency at every single tone position in every single sequencing and noise condition. This would decrease our SNR too much to perform a reliable dipole fit. In order to counterbalance the physical sound properties between constant and random conditions we averaged the obtained auditory evoked fields irrespective of the tone position or the TS frequencies. Considering the tonotopic organization of the auditory cortex [29]–[31], this procedure probably blurred the source localization of the N1m responses, disallowing any statement on subtle location differences.

As described before, in silent background we found differences between sequencing conditions and tone positions regarding the source strength, but not the latency values. In the noisy background the opposite hold true: while there are no differences regarding the source strength, the latency pattern shows a clear drop between first and second tone in the constant condition, but not in the random sequencing condition. It has been shown in several psychophysical studies that cueing a tone with a tone of the same frequency (sometimes termed “iconic cueing”) facilitates the active detection of this tone [2], [5], [32]. Using an active detection task in noise, Okamoto and colleagues [33] showed that reaction times were faster with constant sequencing, and that shorter N1m latencies went along with faster reaction times. In our previous study [11], we furthermore found hints that this also holds true for involuntary tone detection, i.e. when attention is directed away from the auditory domain. The present study confirmed that spectral cueing via constant sequencing seems to facilitate detection of tones in noise even under distracted conditions. Additionally, we could show that this sequencing effect sets in very fast: the effect could be seen already at the first repetition of the same tone.

Natural sounds elicited by the same source can be characterized -among other features- by the fact that they develop over time and thus do not normally change their spectral content abruptly, but rather gradually. An important task for the auditory system is to assign incoming sound signals to distinct sources and to monitor them. For solving this task it is reasonable to expect sounds coming from the same source to be in the spectral vicinity of the signal previously detected. The course of latency decrease observed in our present data seems to be a correlate of the detection and tracking of regular information even under distracted or pre-attentive conditions. In other words, the incoming information is constantly monitored even if voluntary attention is directed to a different modality. In case of the appearance of a salient and potentially important stimulus, this bottom up mechanism can trigger top down processes that might lead to the allocation of attentional resources to the new event [34], [35]. A stimulus is salient if it is sufficiently different from the context in which it occurs. Therefore, a context has to be established in the first place. In silence, we saw a correlate of the build-up of a context in the rapid decrease of source strength after repeated stimulation by the same stimulus as was evident in the constant sequencing condition. A change in stimulation frequency would have resulted in a stronger activation compared to the preceding context [6]. In noise, we did not see any differences in source strength, probably because of noise induced refractoriness of the auditory neurons which was the same in constant as well as in random sequencing. What we did see though was a shortening of the N1m latency, which indicated the accelerated detection of stimuli of the same frequency as the preceding context. Pre-attentive tracking of non-attended input was recently shown to be visible even in the auditory evoked brain stem response [36], [37]. The authors of those studies reported enhanced activity to stimuli occurring repeatedly on a regular basis on local as well as on global time scales. It is feasible that enhanced processing in an early stage such as the brain stem also propagates to higher stages of the auditory pathway and can eventually be seen as faster processing of stimuli with regular properties in the auditory N1m. The reason that we did not see any latency differences between sequencing conditions in the silent condition probably is a ceiling effect: The detection of a tone of sufficient intensity in a quiet surrounding as reflected in the N1m is very fast, independent of the sequencing. Hence, the constant order did not yield any temporal detection advantages compared to random ordering.

### Conclusion

Our results showed that mainly refractoriness was responsible for N1m source strength decrement after repeated presentation of the identical tonal stimulus in a silent surrounding. In a noisy surrounding, neural refractoriness caused by noise which contained the frequency spectrum of the test stimuli completely evened out any source strength differences that might arise from repeated stimulation. In noise, spectral cueing may play a major role for the tracking of incoming stimuli, even if the auditory input is not attended actively. A stimulus is detected faster at its first repetition already. We interpret these data as a correlate of a bottom-up mechanism that helps to constantly monitor incoming information –this might enable the listener to direct top-down attentional resources to the input, in case a salient and potentially important change occurs.
